# Gleanings from Our Exchanges

**Published:** 1873-04-01

**Authors:** 


Psychology in Paris.—Lasegue has just commenced his course on mental affections in the medical clinic at Paris. The great amphitheater of the Faculty could not seat the whole audience, which numbered over 2,000. It is necessary to go an hour or two earlier to get a seat.—*Paris Letter to Vienna*, Jan. 25, 1873.

A New Method of Treating Retention of Urine and Impassable Stricture. By Furneaux Jordan, F.R.C.S. (*Brit. Medical Journal*, Nov. 9, 1872).—The author states that he has been for a long time considering the possibility and propriety, in cases of retention of urine and impassable stricture, of getting behind the stricture and passing instruments forward—the way the urine runs.

The case is related of a patient who had a distended bladder and dribbling of urine, consequent upon retention. Repeated efforts to pass instruments failed.

Dr. F. then carried the left forefinger and pointed bistoury into the rectum ; the point of the bistoury was then thrust forwards through the anterior median line of the rectum, an inch and a quarter from the anus, into the membraneous part of the urethra, and then drawn forwards for a short distance through the incision. The finger could then be easily passed to the stricture in front, and backwards into the bladder. A bougie was passed forwards through the stricture, and emerged from the end of the penis ; the ivory open end was then cut off and carried by the finger backwards into the bladder. The catheter, through which the urine flowed freely, was allowed to remain twenty-four hours and was then withdrawn and a larger one passed through the meatus, until soon a No. 12 could be passed without difficulty. After the patient had been allowed for a few days to pass the urine by natural efforts, during which a little entered the rectum, the rectal fistula was closed in eight days by drawing off the urine several times in the twenty-four hours, especially before each act of defecation.

The author claims that by thus opening the uretha in front of the apex of the prostate gland, the danger of wounding the peritoneum is avoided—a danger which is present in the ordinary puncture of the bladder by the rectal trocar, for the reason that, in cases of retention, resulting from old stricture, the prostate is apt to be crowded back nearer to the peritoneal cul de sac than it is found to be situated in the normal state of things.

For a full report of this interesting lecture, together with the plates illustrative of the operation which is here advocated, we must refer the reader to the journal from which we have made this brief extract.—*Boston Med. & Surg. Journal.*

Reception of Certain Poisons During Sleep.—A writer in the London *Medical Times*, puzzling over the indubitable fact that sewer-men and "night-cart" men are not so liable to suffer from the specific poisons of the materials with which they have to deal as we might expect, suggests that possibly the body is more ready to receive these poisons *during sleep*, and may repel them during waking hours.

This idea seems well worth keeping in mind by physicians, to be proved or disproved by careful observation. The proclivity to take malarial fevers *by night*, on Southern plantations, may be remembered in this connection.—*Boston Med. & Surg. Jour.*

Monti on Constipation in Children.—Dr. Alois Monti (in the *Wien. Med. Presse*, xiii, 26–28, 1872), contributes an exhaustive paper on this subject. He thinks it due to the defective development of the muscular tissue of the intestines, and to the peculiar form of nourishment at this period. He sums up the various causes:—1. Mechanical impediment, as in congenital deformity, imperforate anus, invagination, hernia, etc.; 2. Defective nourishment, as from congenital defects of the throat, too little secretion of milk, etc.; 3. Faulty nourishment, as from excess of casein or defect of fatty matters in the milk, bringing up by hand, etc.; 4. Deficiency or diminution of the peristaltic movements of the intestines, atrophy, etc.; 5. Diminution of the intestinal secretion, as in long-continued diseases in consequence of anemia. Constipation is further a symptom of diseases of the brain and spinal cord; also a consequence of deficiency of drinks, of the use of astringents, presence of ascarides, fruit-stones, etc.; and, in older children, it arises in consequence of deficient bodily exercise. The cause suggests the remedy—cod-liver oil and enemata of cold water, mineral waters, manna, etc.—*London Med. Rec.*

### Gleanings from Our Exchanges.

### TRANSLATIONS FROM THE FRENCH.

BY DR. BOSWORTH, CHICAGO.

*The Medical Union*, of Canada, gives Dr. Brown-Sequard's almost infallible remedy for epilepsy :

℞.—Potassii iodide, ʒ i.

Potassii bromide, ℥ i.

Ammonii bromide, ʒ iss.

Potassa bi-carb., ℈ ii.

Infusionis columbæ, ℥ vi.

Dose, one teaspoonful three times a day before meals, and three teaspoonfuls in a little water at bed-time. Must push the dose till an eruption of acne appears, unless the remedy is causing sleep, lassitude and weakness of the legs. The patient must not omit the medicine a day, as an attack might occur. A slight purgative every five or six weeks increases the effects of the remedy.

Dr. Brown-Sequard says that epileptics generally badly support iron and quinine; but he recommends strychnia, arsenic, oxide of silver, cod liver oil, cold baths, wine, and good nourishment.

*France Medicale*, of February 12th, 1873, gives the following as having been employed by Dr. Boggs, with much success, in leucorrhœa and blenorrhœa :

℞.—Alcoholic tinct. of iodine, ℥ i.

Acidi carbolici, gtts. vi.

Glycerine, ℥ i.

Aquæ dist., ℥ vi.

The carbolic acid in this prevents the iodine from staining.

Same journal says Prof. Richet, of the Medical School of Paris, treats blenorrhagiac orchitis by first puncturing the scrotum and the vaginal tunic, to allow the serosity in the latter tunic to escape. The patient is then confined to abolute quiet, the testicles being supported by any hard substance placed on the anterior surface of the thighs. The genital parts are covered with an aqueous solution of alcohol; the latter is changed every half hour. By this treatment, recovery occurs in about eight days, which is much sooner than by the old method of leeching and poulticing.

This treatment of Prof. Richet also prevents the development, later, of hydrocele of the vaginal tunic, which, in two-thirds of the cases, has its origin from acute orchitis.

The puncturing of the tunic causes the cessation of the intolerable pain always attending inflammation of the testicles. This shortening of the disease gives much less chance for the seminiferous tubes to be obliterated, which quite often occurs when the epididymis remains in a chronic state of induration, as is frequently the case by the old treatment.

Same journal says : "The Russian government has authorized the Medical Academy of St. Petersburg to accept a donation of 200,000 francs from Mrs. Lidie Rodstvenna, for the foundation of a medical course expressly for ladies."

